# Inferior Alveolar Nerve Canal Segmentation on CBCT Using U-Net with Frequency Attentions

**DOI:** 10.3390/bioengineering11040354

**Published:** 2024-04-05

**Authors:** Zhiyang Liu, Dong Yang, Minghao Zhang, Guohua Liu, Qian Zhang, Xiaonan Li

**Affiliations:** 1 College of Electronic Information and Optical Engineering, Nankai University, Tianjin 300350, China; 2 Tianjin Key Laboratory of Optoelectronic Sensor and Sensing Network Technology, College of Electronic Information and Optical Engineering, Nankai University, Tianjin 300350, China; 3 School and Hospital of Stomatology, Tianjin Medical University, Tianjin 300070, China

**Keywords:** medical image segmentation, convolutional neural network, inferior alveolar nerve, attention mechanism, frequency-domain attention

## Abstract

Accurate inferior alveolar nerve (IAN) canal segmentation has been considered a crucial task in dentistry. Failing to accurately identify the position of the IAN canal may lead to nerve injury during dental procedures. While IAN canals can be detected from dental cone beam computed tomography, they are usually difficult for dentists to precisely identify as the canals are thin, small, and span across many slices. This paper focuses on improving accuracy in segmenting the IAN canals. By integrating our proposed frequency-domain attention mechanism in UNet, the proposed frequency attention UNet (FAUNet) is able to achieve 75.55% and 81.35% in the Dice and surface Dice coefficients, respectively, which are much higher than other competitive methods, by adding only 224 parameters to the classical UNet. Compared to the classical UNet, our proposed FAUNet achieves a 2.39% and 2.82% gain in the Dice coefficient and the surface Dice coefficient, respectively. The potential advantage of developing attention in the frequency domain is also discussed, which revealed that the frequency-domain attention mechanisms can achieve better performance than their spatial-domain counterparts.

## 1. Introduction

Inferior alveolar nerve (IAN) injury is one of the most serious complications in dental implant procedures [1,2,3]. This type of injury can occur at various stages, such as during anesthesia and implant placement. It is, therefore, of paramount importance to identify and mark the IAN canal on the medical images before dental surgeries. By having a clear understanding of the position of the nerve, dentists can take the necessary precautions and make informed decisions to minimize the chances of nerve injuries during dental procedures.

Cone beam computed tomography (CBCT) is an effective tool for dental disease diagnosis [4] and provides high-resolution 3D views for the oral and maxillofacial regions, making it possible for a detailed inspection of teeth, jawbones, and the surrounding structures [5]. Due to the fact that IAN tubes are usually displayed as small dots on CBCT slices and are easily confused with cancellous bone imaging, dentists often find it difficult to clearly identify their precise location. Thanks to their potential ability to process 3D volumes as a whole, deep learning methods have been adopted to segment IAN canals from CBCT images [6,7,8,9,10,11,12,13].

Deep learning technology plays an important role in processing medical images nowadays [10,11,12,14,15,16,17,18]. In medical image segmentation tasks, U-shape network architectures [19,20,21,22,23,24] are the most commonly used in medical image segmentation and have achieved top-ranking accuracy in many tasks. U-shape networks typically employ encoder–decoder structures with dense skip connections between the encoder and decoder layers. Generally speaking, the encoder layers are responsible for extracting semantic information and learning effective representations of the input images. The decoder layers, on the other hand, focus on generating a finer segmentation result by jointly considering semantic information and spatial information. The skip connection enables the decoder layers to jointly process the feature maps from both deeper and shallower encoder layers so as to capture image features at different scales and depths and produce more accurate segmentation results [25,26]. When segmenting the IAN canals, UNet is also the most often adopted architecture and has presented good accuracy in both institutional datasets [8,9,10,11] and public datasets [13].

One drawback of the original UNet is that the encoder feature maps are directly concatenated with the decoder feature maps. As the decoder only focuses on refining the segmentation results, the encoder feature maps include a lot of redundant information, which is useless for decoders. Such irrelevant features impose difficulties for decoders, and therefore attention mechanisms are introduced to emphasize the most relevant features [20,27]. For instance [17], introduces a PAL-Net by incorporating both spatial-wise and channel-wise attention modules at the encoder layers to make the network focus on task-relevant features. Note that the attention maps are usually computed according to the original representation, i.e., the spatial domain, of the feature maps. Simply adopting spatial attention and channel attention using convolution layers may fail to capture long-range dependencies from the image. In conventional image processing techniques, it is equally important to analyze the image in both the spatial domain and the frequency domain. As pointed out in [28], the channel-wise attention in the squeeze–excitation (SE) module is equivalent to that generated according to the direct current (DC) component of each feature map. By taking more frequency components into consideration, the performance can be further improved. However, while innumerable attention methods have been proposed for spatial domain analysis, attention methods on the frequency domain are very limited [28,29].

In this paper, we propose an effective frequency-domain attention module (FAM) for the U-shape network. Although the proposed FAM only includes 56 parameters, the IAN canal segmentation accuracy can be significantly improved. To fairly evaluate the performance, a publicly available IAN canal dataset with accurate segmentation labels is used to train and evaluate the performance [13]. The 91 fully annotated subjects are split into training and test sets with 68 and 23 subjects, respectively, following the same splitting strategy as [13]. The experiment results reveal that our proposed method is able to achieve a Dice coefficient of 75.55%. Compared to UNet, our proposed method presents a significant improvement in segmentation accuracy by adding only 224 parameters, which highlights the effectiveness of introducing frequency-domain attention mechanisms in medical image segmentation tasks.

## 2. Related Work

### 2.1. U-Shape Networks

Despite the fact that DeepLab networks [30,31,32] have achieved tremendous performance in many image segmentation tasks by employing a pre-trained backbone, U-shape networks are still considered as the most efficient network structure in medical image segmentation tasks due to the lack of effective pre-trained backbones. Originally proposed in [19], UNet adopted a symmetric encoder–decoder structure, with skip connections between the encoder and decoder layers. Such structure enables it to efficiently learn the features even with limited training samples, and therefore has become increasingly popular in biomedical segmentation tasks [33,34].

To further improve segmentation accuracy, many useful modifications have been made to the original UNet, where most efforts focus on improving the efficiency of the skip connections. For instance, UNet++ [21] and UNet3+ [22] focus on introducing more skip connections between different levels of encoder and decoder layers. By incorporating a squeeze–excitation module [35] at the skip connection, DSEU-net [27] utilized the channel attention mechanism and reweighted the feature maps of different channels. Attention UNet [20], on the other hand, introduced a special attention mechanism at the skip connection to enforce the decoder, focusing more on features in a specific region.

In addition, UNet’s performance is also expected to improve by designing better encoders. Inspired by Transformer’s great success in natural language processing [36], network architectures with Transformer-type encoders have also attracted extensive attention [37,38,39,40]. Despite that the Transformer encoder enables them to capture long-range dependencies and therefore improve segmentation accuracy, these architercutres still adopted skip connections to transfer detail information to the decoder.

In general, the skip connections have been proven to efficiently transfer the spatial information from the encoder so that the decoder can fuse both the spatial and semantic information to obtain a finer segmentation. However, directly fusing information from the encoder with information from the decoder may not be an efficient approach, as too much redundant information is also transferred to the decoder. In [41], by adding a high-pass filter at the skip connection, the contrast attention UNet (CAUNet) is able to further improve segmentation accuracy in kidney segmentation tasks without introducing any parameters.

### 2.2. Attention Mechanism

Attention mechanisms enable the network to selectively focus on important features or regions so as to make more accurate and context-aware predictions. When applied to image processing tasks, channel attention and spatial attention are mostly considered. For instance, DSEU-net [27] adopted the channel attention mechanism to emphasize the more important feature map channels. Attention UNet [20] introduced the spatial attention mechanism to focus on the important regions. The attention mechanisms can also be simultaneously applied channel-wise and spatial-wise [42,43,44,45], which is expected to make the networks focus on the important regions within the feature map channel.

When segmenting small objects, spatial attention mechanisms are usually adopted [46], as they filter out the large but irrelevant regions and force the decoder to emphasize the small objects. Note that the spatial attention maps are usually generated using convolution layers directly on the feature maps, which capture the dependencies of neighboring regions. If we expect to capture the long-range dependency, large convolution kernels are generally required, which significantly increases the number of parameters.

In conventional image processing, it is well known that a filter can be designed either in the spatial domain or in the frequency domain. Spatial attention, which generates a weighting map and element-wise multiplies it by the feature maps, can be regarded as a filter defined in the spatial domain. This motivates us to investigate ways to adopt spatial attention while designing a filter on the frequency domain.

In fact, some attention mechanisms can also be treated as frequency attention mechanisms. It has been proven in [28] that the global average pooling in the SE module is equivalent to extracting only the direct current (DC) component from the feature maps. To further improve the performance of the SE module, FcaNet proposed to retain multiple low-frequency components on the frequency-domain map and perform frequency attention mechanisms by applying channel-wise attention [28]. By noting that the frequency components in the FcaNet are manually selected by experiments, we propose to develop a more general frequency attention mechanism that adaptively determines which frequency components to emphasize. As we will show in this paper, by adding only 224 parameters to UNet, the segmentation accuracy can be significantly improved.

## 3. Frequency Attention UNet

**Figure 1:**
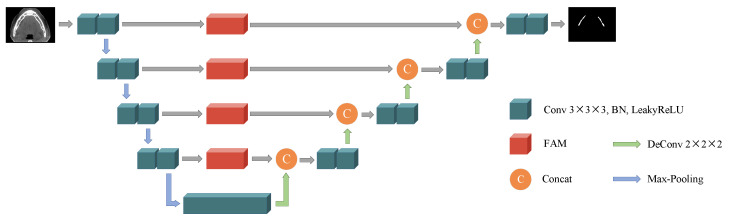
Architecture of our proposed FAUNet. The FAUNet generally has a similar structure, while an FAM is added at each skip connection.

In this paper, we propose a frequency attention UNet (FAUNet), as shown in Figure 1. The proposed FAUNet has basically a similar structure as a conventional UNet, while a frequency attention module (FAM) is added at each skip connection. In our proposed FAUNet, the feature maps from the encoder layers are first reweighted in the frequency domain by the FAMs before concatenating with the decoder feature maps. The FAM generates an attention map, applied to the feature maps in the frequency domain, and filters out the irrelevant features from the encoder feature maps. Before introducing the motivations and the structure of the proposed FAM in detail, the fundamentals of the spatial-frequency-domain transform will first be revisited.

### 3.1. Discrete Cosine Transform Revisit

The frequency-domain image of the feature map can be obtained through discrete cosine transform (DCT) [47]. For a spatial-domain function f(x,y,z), its DCT is defined as

(1) $$F\left(u,v,w\right)=\alpha \left(u\right)\alpha \left(v\right)\alpha \left(w\right)\sum_{x=0}^{H-1}\sum_{y=0}^{W-1}\sum_{z=0}^{D-1}f\left(x,y,z\right)B_{u,v,w}^{x,y,z},$$

where $B_{u,v,w}^{x,y,z}$ is the basis function, which is defined as

(2) $$B_{u,v,w}^{x,y,z}=\cos\left(\frac{\pi u}{H}\left(x+\frac{1}{2}\right)\right)\cos\left(\frac{\pi v}{W}\left(y+\frac{1}{2}\right)\right)\cos\left(\frac{\pi w}{D}\left(z+\frac{1}{2}\right)\right).$$

α(u), α(v), α(w) are the normalization constants that ensure the orthogonality of the basis. As (1) shows, different from the discrete Fourier transform (DFT), which also generates the frequency-domain representation of a discrete signal, the DCT representation of a signal is composed of real-valued numbers, making it easier to be processed for neural networks.

**Figure 2:**
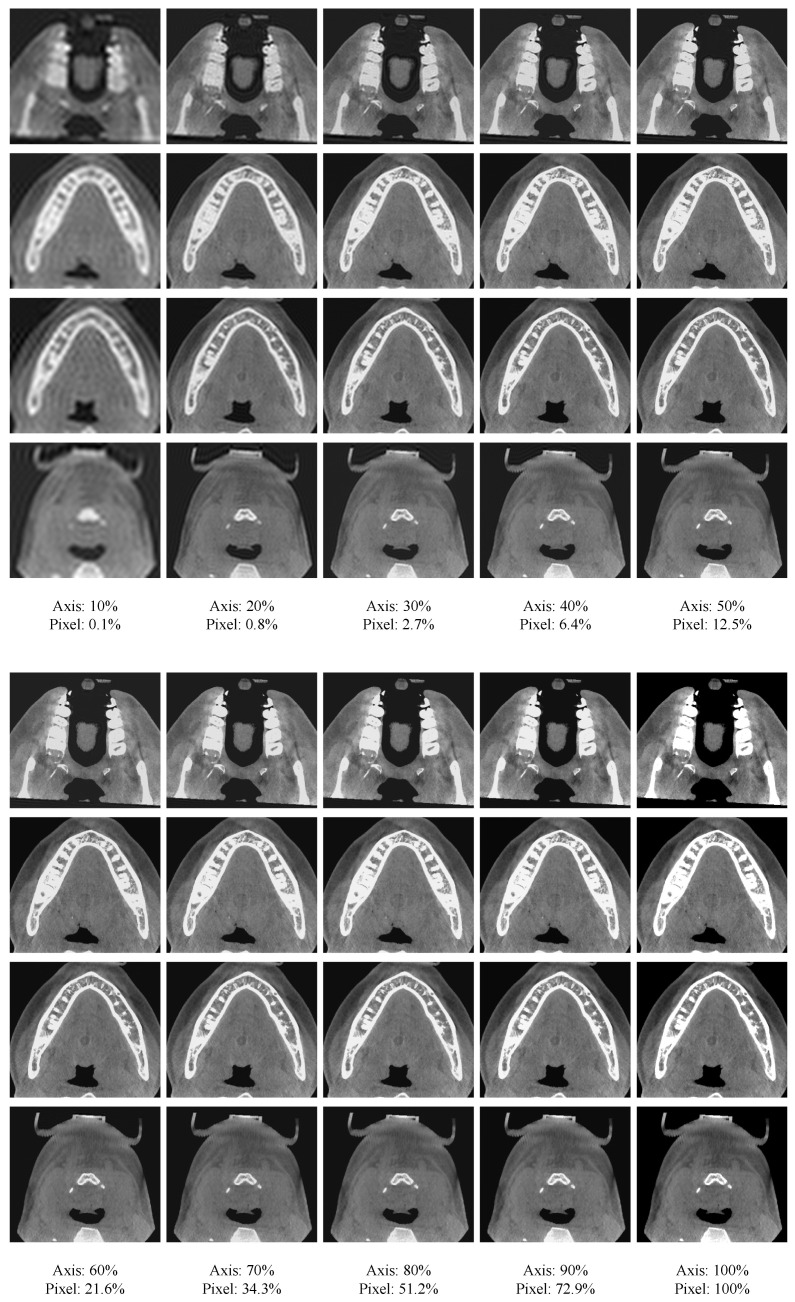
Reconstruction of spatial-domain images by preserving different levels of frequency components. Axis means the proportion of low-frequency components retained in each axis direction. Meanwhile, pixel denotes the proportion of retained low-frequency components compared to all frequency components.

An important observation from the DCT is that it usually concentrates the most energy at the upper-left corner in the frequency domain, which implies that the image content is mostly determined by lower frequency components. For instance, Figure 2 presents the reconstruction result of 2D CBCT slices using different proportions of frequency components. From Figure 2, we can see that by retaining a small proportion of frequency components, most details of the image can be recovered. As the retaining proportion increases, more details of the images can be retained, and the recovered image gradually becomes sharper and clearer. Such an observation suggests that the high-frequency components can be suppressed to generate a more compact representation of the features.

**Figure 3:**
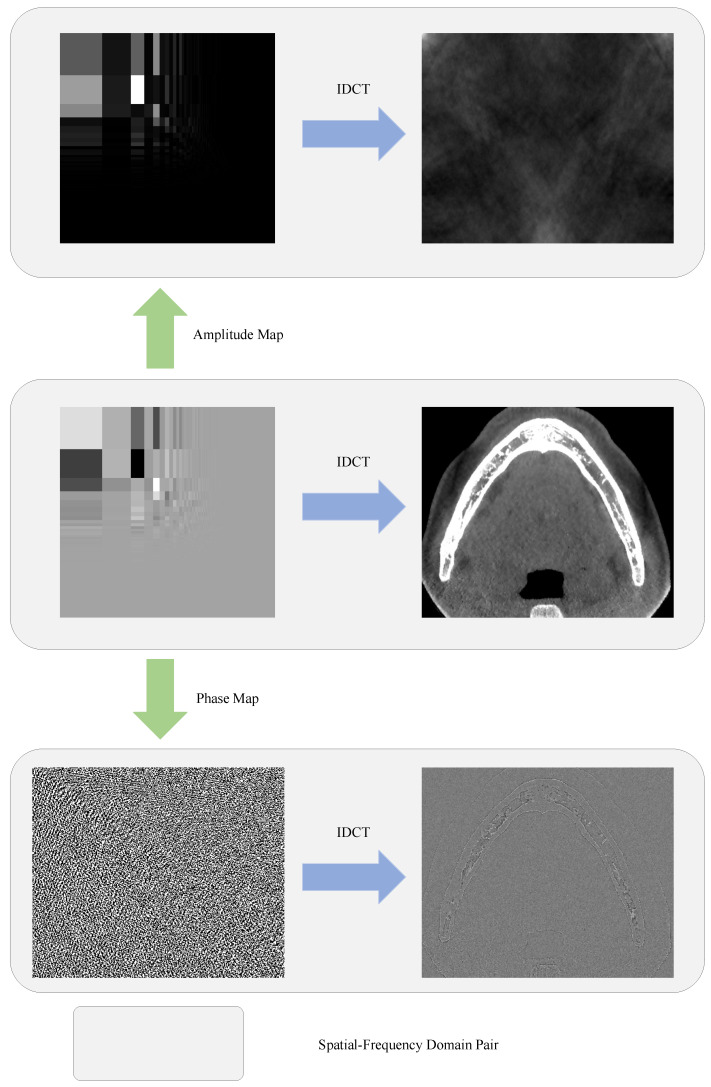
Visualized examples of recovered CBCT slice from its DCT representation using IDCT on different DCT components. Top row: recovered from amplitude map only. Middle row: recovered from both amplitude and phase maps. Bottom row: recovered from phase map only. In the first two rows, the frequency-domain representations are presented in logarithmic coordinates for better visualizing the amplitude distribution.

Another observation from the DCT is the different roles that the amplitude map and phase map play, where the phase is defined as the sign of a frequency component. Figure 3 presents the reconstructed images from either the amplitude or the phase. It can be clearly seen from Figure 3 that the spatial information of the pixels is mainly determined by the phase, while the style of the image is mainly determined by the amplitude map. This motivated us to develop an attention mechanism from the aspect of the frequency domain, which not only reweights the amplitudes but also modulates the phases, in order to filter out the irrelevant features.

### 3.2. Frequency Attention Module

**Figure 4:**
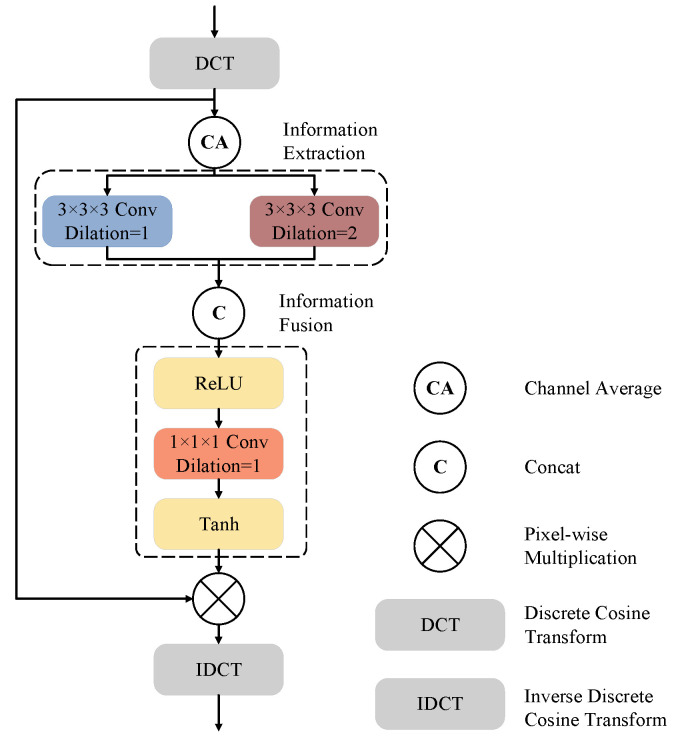
The architecture of the proposed FAM block.

Figure 4 presents the design of our proposed FAM. We call it frequency-domain attention, as all operations are performed in the frequency domain using DCT. Our proposed FAM mainly consists of two stages: the information extraction (IE) stage and the information fusion (IF) stage. The input feature maps are first transformed to the frequency-domain representation using DCT and then processed by a channel-wise average pooling operation to obtain the mean frequency-domain features before being fed to the IE and IF stages.

In the IE stage, convolution layers with kernel sizes of 3×3×3 and different dilation rates are adopted to interact between different frequency components. The convolution layer with a dilation rate of R=1 allows interaction between the adjacent frequency components, while the convolution layer with a dilation rate of R=2 enables interaction only among the components whose frequencies have the same parity (i.e., odd or even). In the IF stage, the feature maps are first channel-wise concatenated and then activated using ReLU. A convolution layer with a kernel size of 1 is employed to fuse the information from both branches of the IE stage, after which the tanh function is adopted to generate the frequency-domain attention map. The reason that we use tanh instead of sigmoid, as most attention mechanisms do, is that both the amplitudes and the phases should be adaptively tuned by the attention map. As we will show in our experiments, modulating the phases is beneficial in improving segmentation accuracy. After obtaining the attention map, the frequency-domain representation of the input feature maps is multiplied by the attention map and then converted back to the spatial domain using inverse discrete cosine transform (IDCT).

The number of parameters in the proposed FAM is very limited. More specifically, the number of parameters of an FAM can be computed as

(3) $$\begin{array}{cc} P_{FAM} & =\underset{kernel}{\underset{︸}{\;3^{3}\;}}\times \underset{C_{in}}{\underset{︸}{\;\;1\;\;}}\times \underset{C_{out}}{\underset{︸}{\;\;1\;\;}}\times \underset{N}{\underset{︸}{\;\;2\;\;}}+\underset{kernel}{\underset{︸}{\;1^{3}\;}}\times \underset{C_{in}}{\underset{︸}{\;\;2\;\;}}\times \underset{C_{out}}{\underset{︸}{\;\;1\;\;}}\times \underset{N}{\underset{︸}{\;\;1\;\;}}=56 \end{array}$$

where kernel denotes the number of parameters of a convolution kernel and $C_{in}$ and $C_{out}$ are the numbers of input channels and output channels, respectively. *N* is the numbers of the convolution layer. As shown in Figure 1, our proposed FAUNet is modified from UNet by adding four FAMs at the skip connections. Therefore, our proposed FAUNet has only 224 more parameters than a UNet.

### 3.3. Loss Function

The loss function we adopted is the sum of the cross entropy loss and Dice loss, i.e.,

(4) $$L=L_{Dice}+\lambda L_{CE},$$

where λ is the tradeoff coefficient, which is set to be 1 in this paper. $L_{Dice}$ and $L_{CE}$ denote the Dice loss and cross entropy loss, respectively, and are given as

(5) $$\begin{array}{c} L_{Dice}=1-\frac{2\sum_{i=1}^{N}p_{i}g_{i}}{\sum_{i=1}^{N}p_{i}^{2}+\sum_{i=1}^{N}g_{i}^{2}} \end{array}$$

and

(6) $$L_{CE}=-\sum g_{i}log\left(p_{i}\right),$$

where $p_{i}$ and $g_{i}$ are the predicted probability and the ground truth of the *i*-th voxel, respectively.

### 3.4. Evaluation Metrics

In this paper, the volumetric symmetric metric, i.e., the Dice coefficient, and the boundary accuracy metrics, including the surface Dice coefficient (SD), the 95% Hausdorff distance (HD95), and the average symmetric surface distance (ASSD), are used to evaluate the segmentation accuracy.

In particular, by denoting *A* and *B* as two binary segmentation maps, the Dice coefficient is defined as

(7) $$Dice=\frac{2\times |A\cap B|}{\left|A|+|B\right|},$$

where |A| denotes the area of foreground voxels on *A*.

Similarly, SD measures the similarity of two boundaries. By denoting $S_{A}$ and $S_{B}$ as the boundaries of the segmentation maps *A* and *B*, respectively, SD is defined as

(8) $$SD=\frac{2\times |S_{A}\cap S_{B}|}{|S_{A}|+|S_{B}|}.$$

The Hausdorff distance measures the maximum distance of a set to the nearest point in the other set. In this paper, to eliminate the prominant influences of the outlier points, a 95% Hausdorff distance is adopted to measure the surface accuracy, which is defined as

(9) $$HD95\left(A,B\right)=\max\left(K_{a\in A}^{95th}\left(\min_{b\in B}d\left(a,b\right)\right),K_{b\in B}^{95th}\left(\min_{a\in A}d\left(b,a\right)\right)\right),$$

where d(·,·) denotes the Euclidean distance between two points. $K_{a\in A}^{95th}$ means the max Euclidean distance when taking the 95% percentile distances into consideration.

The ASSD evaluates the average symmetric surface distance between two images, taking into account the symmetry of the images and averaging the distances between different surfaces to provide a more comprehensive evaluation of segmentation accuracy. The ASSD is defined as

(10) $$ASSD=\frac{\sum_{a\in A}\min_{b\in B}\left\{d\left(a,b\right)\right\}+\sum_{b\in A}\min_{a\in B}\left\{d\left(b,a\right)\right\}}{\left|A|+|B\right|}.$$

## 4. Experiment Results

### 4.1. Data

In this paper, a publicly available IAN canal segmentation dataset [13] is used to evaluate our proposed method. The dataset consists of the dental CBCT of 347 subjects, where 91 of them are with dense 3D annotations and 256 are with sparse 2D annotations. In this paper, we only use the subjects with dense annotations and split the 91 subjects following the same allocation as described in [13], where 68 samples are used as the training set and 23 samples are used as the testing set. All samples in the dataset have a uniform voxel size of 0.3×0.3×0.3 mm^3^, while their matrix sizes range from 151×265×369 to 171×396×463.

### 4.2. Implementations

The experiment is conducted on a workstation with NVidia TITAN RTX GPU, and the proposed method is implemented using PyTorch 2.0.0 and monai 1.1.0.

Before training, the intensities of the images are first clipped to the range of [−300,800] HU and then normalized to zero mean and unit variance. During training, the images are randomly cropped to patches of size 112×112×112 before being fed to the network. AdamW [48] is adopted as the optimizer during training, and the initial learning rate is 0.0001. The learning rate decays at the end of each epoch using a polynomial decay scheduling, where the learning rate at the *t*-th epoch is given as

(11) $${\mathrm{lr}}^{\left(t\right)}={\mathrm{lr}}_{\mathrm{init}}\left(1-\frac{t}{t_{\max}}\right),$$

where ${\mathrm{lr}}_{\mathrm{init}}$ and ${\mathrm{lr}}^{\left(t\right)}$ are the initial learning rate and the learning rate at the *t*-th epoch, respectively. $t_{\max}$ denotes the number of epochs to be trained. In this paper, we define an epoch as 100 update iterations, and the model is trained for 500 epochs. Data augmentation techniques, including elastic deformation, random scaled zooming, random flipping, random contrast adjustment, and random scaled intensity, are also adopted during training. The details of our data augmentation techniques are summarized in Table 1.

### 4.3. Results

In this section, the segmentation accuracy of our proposed method on the testing set is presented. For the sake of comparison, several other U-shaped networks, including UNet [19], SEU-net [27], Attention UNet [20], UNet++ [21], UNet3+ [22], TransUNet [37], and UNETR [38], are also trained on the same training set.

**Figure 5:**
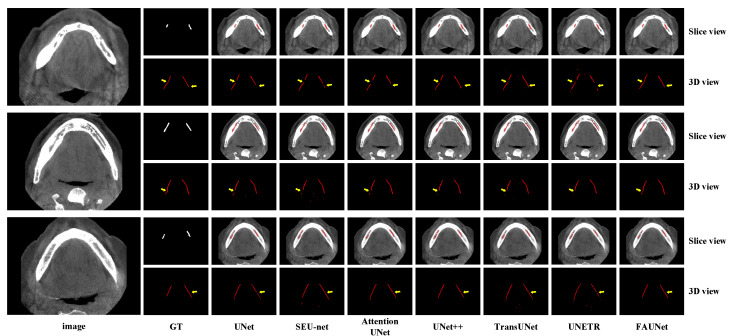
Visualized examples of the segmentation results by various methods. For more clear visualization, the segmentation results are presented in both slice view and 3D view.

Figure 5 shows several segmentation examples of the testing set. As the IAN canals are tubes that span across many 2D slices, the 3D views of the IAN canals are also plotted in Figure 5 to give a more complete view of the segmentation result. As we can see from Figure 5, the segmentation results of UNet, SEU-net, UNet++, and UNet3+ are corrupted by discontinuities on the segmented IAN canals due to the fact that the convolution operations are performed in the spatial domain using convolution kernels with limited fields of view. Therefore, when segmenting small objects, such as IAN canals, it becomes more difficult to determine whether a voxel belongs to the foreground or background when only considering the local features. For the Transformer-based methods, such as TransUNet and UNeTR, despite the fact that the Transformer encoders are good at capturing long-range dependencies, they need more training samples to train the network due to the overwhelmingly large number of parameters. Our proposed FAUNet, on the other hand, is able to capture long-range dependencies using the attention mechanism in the frequency domain, leading to better segmentation accuracy.

Numerical results also validate our observations. Table 2 presents the evaluation results of all the methods mentioned on the test set. The segmentation results reported by the data producer [13] are also listed in Table 2 for the sake of comparison. Note that, despite the fact that we use the same UNet structure as [13], our experiment suggests a higher Dice coefficient in Table 2 thanks to the richer data augmentations in our experiment. As we can see from Table 2, our proposed FAUNet achieved the best performance on all evaluated metrics. UNet, on the other hand, also achieved good results compared to the other UNet variants, which coincides with the observations in nnUNet [49] that UNet is able to achieve top-ranking performance by using properly designed training strategies. It is also interesting to see from Table 2 that UNETR and TransUNet, which adopt Transformer encoders, do not perform well in segmenting IAN canals. As we can see from Table 2, both UNETR and TransUNet have a much larger number of parameters, making it difficult to be trained, especially with a limited sized training set.

As we have analyzed in Section 3.2, our proposed FAM only includes 56 parameters, which means that our proposed FAUNet has only 224 more parameters than UNet. As Table 2 shows, our proposed FAUNet is able to significantly improve segmentation accuracy by adding a very limited number of parameters, which highlights the efficiency of our proposed frequency-domain attention mechanism.

## 5. Discussion

### 5.1. Spatial-Domain Attention versus Frequency-Domain Attention

Note that most attention mechanisms focus on producing attention directly on the feature maps. To demonstrate the effectiveness of the frequency-domain attention, we chose to modify Attention UNet and SEU-net by producing attention maps on the frequency domain. Moreover, the performance of a modified FAUNet by removing the DCT is also evaluated. The evaluation results are presented in Table 3.

As Table 3 shows, among all evaluated methods, the frequency-domain attention methods, i.e., “Attention UNet + DCT”, “SEUNet + DCT”, and “FAUNet”, presented prominent improvements over their spatial-domain counterparts. Compared to the spatial-domain attention mechanisms, the corresponding frequency-domain attention mechanism achieved Dice coefficient improvements of 0.78%, 0.55%, and 0.83% in Attention UNet, SEU-net, and our proposed FAUNet. This suggests that generating attention mechanisms from the frequency domain would provide additional benefits without adding any tunable parameters.

### 5.2. Ways to Generate Frequency-Domain Attention

**Figure 6:**
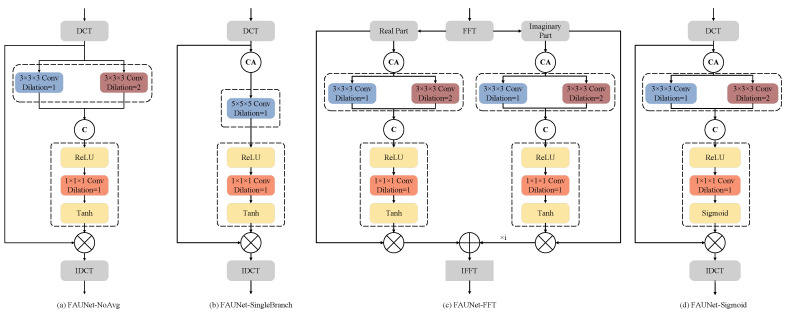
Structures of the various frequency-domain attention methods.

The proposed FAM simultaneously applies attention to the amplitude and phase of the frequency-domain representation of the feature maps, and the frequency-domain representation is obtained by applying DCT to the feature maps. To validate the effectiveness of the layers included in our proposed FAM, ablation experiments are performed in this subsection. The numerical evaluation results are presented in Table 4. The modified FAMs are depicted in Figure 6. In particular, “FAUNet-NoAvg” denotes the network by removing the channel-wise average pooling in the FAM. “FAUNet-SingleBranch” denotes that the convolution layer with a dilation rate of R=2 is removed in the FAM while the kernel of the convolution layer with a dilation rate of R=1 is expanded to 5×5×5. “FAUNet-FFT” denotes that fast Fourier transform (FFT) is adopted to obtain the frequency-domain representations, where the real and imaginary parts of the FFT representations are separately processed. “FAUNet-Sigmoid” denotes that the tanh in the FAM is replaced by a sigmoid function, which means that the phase of the DCT maps will not be modulated and the attention is only adopted to modulate the amplitude.

It can be observed from Table 4 that the attention map generated by tanh is 0.66% better than that generated by sigmoid activation. This identifies a clear distinction between the frequency-domain and spatial-domain attention mechanisms in that it is important to simultaneously modulate the amplitude and phase, which also validates the observations discussed in Section 3.1 that the phase map includes critical information about the original images.

By comparing the performance of double-branch FAM and single-branch FAM, we can see from Table 4 that the FAUNet achieved a 1.01% improvement in the Dice score compared to the FAUNet-SingleBranch while reducing 56% of the parameters. The channel average operation also plays an important role in the attention mechanism. By applying a channel average operation, the network received a 1.45% gain in terms of Dice. The results suggest that using the average frequency-domain representation of all channels can yield a more stable performance. Moreover, DCT, which generates real-valued frequency-domain representations, presented better performance in generating the frequency-domain attention mechanisms.

Moreover, the impact of different spatial-frequency-domain transformation methods on model performance is also discussed. Despite the fact that FFT is a more commonly adopted approach for spatial-frequency transform, our experiments suggest that using FFT instead of DCT leads to much worse performance, which should be blamed on the resulting phase ambiguities and energy concentration. As we have discussed in Section 3.1, the phase of DCT is determined by the sign and only takes two values, i.e., {0,π}. For FFT, however, the phase could be drawn from [0,2π), which is much more difficult to tune compared to the DCT case. As a result, in our experiments, FAUNet-FFT achieves much worse performance than our proposed FAUNet, which uses DCT for spatial-frequency-domain transform.

### 5.3. Analysis of Attention Maps

**Figure 7:**
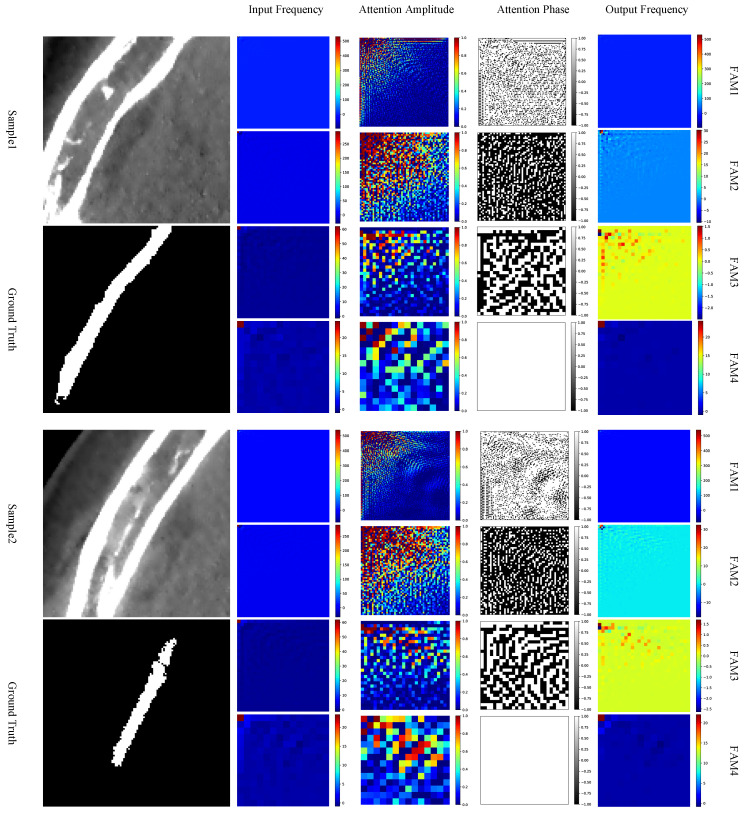
Visualized examples of the attention maps in FAM at different depths of skip connections. For each sample, the columns from left to right denote the DCT representation of the input, amplitude of the attention map, phase of the attention map, and the DCT representation of the FAM output, respectively. Best viewed in color.

It is also interesting to take a closer look at the frequency-domain attention maps generated by the FAM. By randomly selecting two input patches with foreground from the test set, the visulized results of the amplitude and phase maps of the generated attention maps of the sample patches are depicted in Figure 7.

From the attention amplitude maps, we can see that components with lower frequencies, i.e., near the upper-left corner, are assigned to higher weights, while those with higher frequencies are assigned to lower or even zero weights. Such an observation coincides with the DCT property of images, where the energy in the frequency domain is concentrated at lower frequencies, as we have revisited in Section 3.1. When observing the attention phase maps, however, there is no obvious tendency shown on the phase maps. It is also very interesting to see that, for the examples in Figure 7, at FAM4, i.e., the FAM at the deepest skip connection, there is no phase inversion at all.

**Figure 8:**
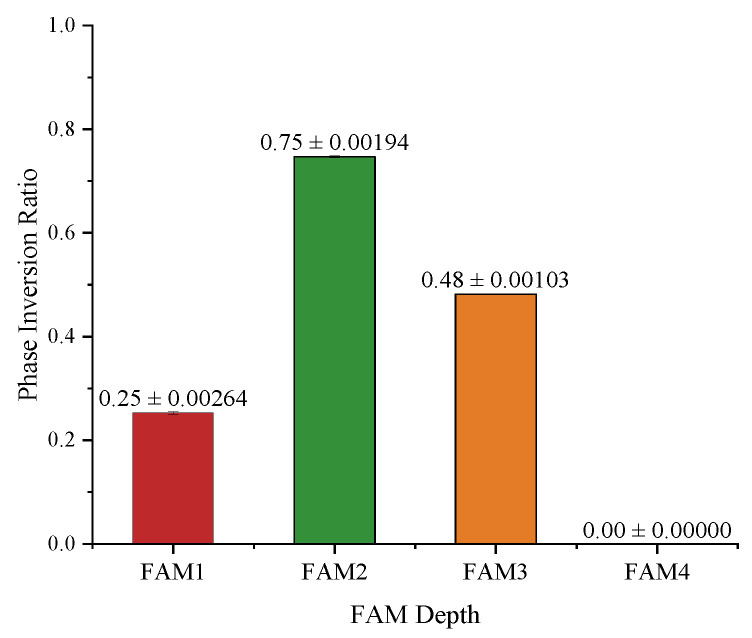
Statistics on phase inversion ratio presented in the FAM attention maps.

To analyze whether the ratio of phase inversion is related to the depth where the FAM is installed, we counted the number of phase inversions across all subjects in the test set, as shown in Figure 8. As we can see, the ratio of phase inversion is closely dependent on the depths of the FAMs. There is no phase inversion at the deepest FAM, i.e., FAM4. FAM2, on the other hand, has the largest proportion of phase inversion, with about 75% of the phases inverted. As the phases have a significant impact on the texture of an image, such observations in turn support the intuition of UNet that deeper skip connections contribute more to semantic information and shallower skip connections contribute more to spatial information, generating a finer segmentation.

**Figure 9:**
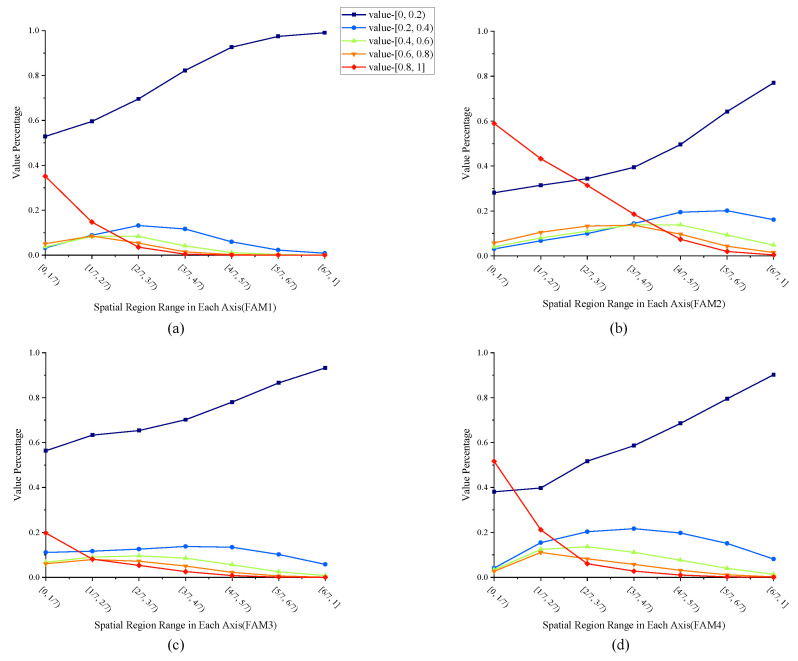
Statistics on amplitude weights on the attention maps of FAMs. (**a**–**d**) present the statistics of the amplitude weights on attention maps of FAM 1-4, respectively. Each FAM attention map is first divided into 7 non-exclusive regions, $F_{i}=\left\{A\left(u,v,w\right)|u\leq \frac{i}{7}H,v\leq \frac{i}{7}W,w\leq \frac{i}{7}D\right\}$, where *A* is the attention map and (H,W,D) is the shape of the attention map. Then, the amplitude of the elements within the regions $S_{i}=F_{i}/F_{i-1}$ is considered.

Figure 9 further shows the statistics of the amplitudes in the attention maps on the entire test set. The blue lines, which represent the proportion of amplitudes within the range of [0,0.2), are typically high in all FAMs. This suggests that our proposed frequency-domain attention mechanism suppresses a large proportion of frequency components and therefore reduces the overwhelming information in the feature maps. We can also observe from Figure 9 that the red line, which represents the proportion of amplitudes within the range of [0.8,1], reduces for higher-frequency components. This suggests that our proposed frequency-domain attention exhibits a preference to give low-frequency components higher weights. The statistical results indicate that, in frequency-domain attention, the network tends to preserve low-frequency information to capture the main content while altering the phase of frequency components in shallow layers that contain rich, detailed information to obtain edge information, which also supports our hypothesis in Section 3.1.

## 6. Conclusions

In this paper, we have proposed a frequency-domain attention mechanism to improve segmentation accuracy. By inserting the proposed FAM at the skip connections of UNet, our proposed FAUNet has presented a significant improvement in segmenting the IAN canal from the CBCT by adding a negligible number of parameters. The effectiveness of frequency-domain attention is also discussed. Our experiments reveal that, by directly converting other popular spatial attention mechanisms to the frequency domain using DCT, their segmentation accuracy can also be improved, which highlights the great potential of adopting frequency attention mechanisms in medical image segmentation tasks. Note that, due to the limited number of samples in the publicly available IAN dataset, the performance of our proposed FAUNet on large datasets remains unverified. It is, therefore, of paramount importance to investigate the effectiveness of frequency-domain attention mechanisms on larger and more diverse datasets, which will be conducted in our future work.

## Figures and Tables

**Table 1:** Data augmentation methods applied during training.

Method	Probability	Settings
Elastic deformation	0.3	σ∼[0.005,0.01], M∼[0.005,0.01]
Zooming	0.3	zooming scale factor ∼[0.8,1.2].
Rotation	0.3	rotation angle ∼[−π,π] for each plane.
Axis flip	0.5 (each axis)	\
Contrast adjustment	0.2	γ=(0.7,1.5)
Scale intensity adjustment	0.2	factors ∼[−0.1,0.1]

**Table 2:** Numerical evaluation results of UNet, some UNet variants, and our proposed FAUNet on the test set. The number of parameters and the computational complexity are also presented. The most prominent result for each column is highlighted in bold font.

Method	Dice (%)	HD95	ASSD	SD (%)	Params (M)	FLOPs (G)
UNet [13] *	67.00	/	/	/	/	/
UNet	73.16	22.05	3.60	78.53	22.93	363.5
Attention UNet	72.60	26.03	3.63	78.07	23.02	366.5
SEU-net	71.94	40.13	6.28	76.19	25.29	436.3
UNet++	74.10	16.99	2.65	79.74	26.64	1282.3
UNet3+	69.78	19.83	3.00	75.36	20.40	2622.8
UNETR	55.99	95.73	12.08	54.61	101.62	419.6
TransUNet	71.53	37.03	5.53	75.61	68.47	272.4
FAUNet	**75.55**	**16.98**	**2.63**	**81.35**	22.93	363.6

* Only Dice coefficient is reported in [13].

**Table 3:** Numerical evaluation results of networks with spatial-domain and frequency-domain attention mechanisms on the test set. The most prominent result for each column is highlighted in bold font.

Method	Dice (%)	HD95	ASSD	SD (%)
Attention UNet	72.60	26.03	3.63	78.07
Attention UNet + DCT	73.38	29.31	4.44	78.14
SEU-net	71.94	40.13	6.28	76.19
SEU-net + DCT	72.49	21.08	3.31	77.69
FAUNet − DCT	74.72	18.11	2.80	80.16
FAUNet	**75.55**	**16.98**	**2.63**	**81.35**

**Table 4:** Evaluation results of different frequency-domain attention approaches on the test set. The most prominent result for each column is highlighted in bold font.

Method	Dice (%)	HD95	ASSD	SD (%)
UNet	73.16	22.05	3.60	78.53
FAUNet	**75.55**	16.98	**2.63**	**81.35**
FAUNet-NoAvg	74.10	20.47	3.14	79.09
FAUNet-SingleBranch	74.54	17.59	2.75	79.92
FAUNet-Sigmoid	74.89	**16.56**	2.93	80.41
FAUNet-FFT	74.70	19.58	2.94	79.89

## Data Availability

The original data presented in the study are openly available in https://ditto.ing.unimore.it/maxillo/ (accessed on 21 February 2024).
